# Strong Electronic Interaction Enhanced Electrocatalysis of Metal Sulfide Clusters Embedded Metal–Organic Framework Ultrathin Nanosheets toward Highly Efficient Overall Water Splitting

**DOI:** 10.1002/advs.202001965

**Published:** 2020-09-21

**Authors:** Ming Zhao, Wei Li, Junying Li, Weihua Hu, Chang Ming Li

**Affiliations:** ^1^ Key Laboratory of Luminescence Analysis and Molecular Sensing (Southwest University) Ministry of Education Institute for Clean Energy and Advanced Materials School of Materials and Energy Southwest University Chongqing Key Laboratory for Advanced Materials and Technologies of Clean Energies Southwest University Chongqing 400715 China; ^2^ Institute of Materials Science and Devices School of Materials Science and Engineering Suzhou University of Science and Technology Suzhou 215009 China; ^3^ Institute of Advanced Cross‐field Science College of Life Science Qingdao University Qingdao 200671 China

**Keywords:** electronic interactions, metal–organic frameworks, metal sulfide clusters, ultrathin nanosheets, water splitting

## Abstract

Unique metal sulfide (MS) clusters embedded ultrathin nanosheets of Fe/Ni metal–organic framework (MOF) are grown on nickel foam (NiFe‐MS/MOF@NF) as a highly efficient bifunctional electrocatalyst for overall water splitting. It exhibits remarkable catalytic activity and stability toward both the oxygen evolution reaction (OER, *ƞ* = 230 mV at 50 mA cm^−2^) and hydrogen evolution reaction (HER, *ƞ* = 156 mV at 50 mA cm^−2^) in alkaline media, and bi‐functionally catalyzes overall alkaline water splitting at a current density of 50 mA cm^−2^ by 1.74 V cell voltage without *iR* compensation. The enhancement mechanism is ascribed to the impregnated metal sulfide clusters in the nanosheets, which not only promote the formation of ultrathin nanosheet to greatly enlarge the reaction surface area while offering high electric conductivity, but more importantly, efficiently modulate the electronic structure of the catalytically active atom sites to an electron‐rich state via strong electronic interaction and strengthen the adsorption of oxygenate intermediate to facilitate fast electrochemical reactions. This work reports a highly efficient HER/OER bifunctional electrocatalyst and may shed light on the rational design and synthesis of uniquely structured MOF‐derived catalysts.

## Graphical abstract

Fe/Ni‐based metal–organic framework ultrathin nanosheets with embedded metal sulfide clusters exhibit remarkable catalytic activity and stability toward both the oxygen evolution reaction and hydrogen evolution reaction in alkaline media. The metal sulfide clusters efficiently modulate the electronic structure of the catalytically active layer to an electron‐rich state via strong electronic interaction to facilitate the electrochemical reactions.

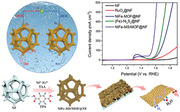

## 1 Introduction

Water electrolysis is an economically feasible and environment‐friendly strategy for large‐scale production of clean hydrogen energy.^[^ ^1^, ^2^, ^3^, ^4^ ^]^ Highly efficient electrocatalysts are required to essentially reduce the overpotential and accelerate the sluggish hydrogen evolution reaction (HER) and oxygen evolution reaction (OER) for improved overall energy efficiency in water electrolysis.^[^ ^1^, ^5^, ^6^ ^]^ Pt‐ and Ir/Ru‐based noble metals are the best catalysts for HER and OER, respectively.^[^ ^7^, ^8^, ^9^ ^]^ Unfortunately, their large‐scale applications are hampered by their high cost and scarcity. As a consequence, it is extremely necessary to develop highly active while sustainable electrocatalysts for HER and OER.^[^ ^10^, ^11^ ^]^

As a type of blooming star materials, metal–organic frameworks (MOFs) formed by metal nodes and organic ligands have attracted extensive attention because of their well‐defined while highly tunable compositions and structures in recent years.^[^ ^12^, ^13^, ^14^ ^]^ In electrocatalysis, MOFs were used as precursors and/or sacrificial templates to prepare various electrocatalysts including metal oxides, phosphides, and sulfides supported on porous carbon through pyrolysis under different conditions.^[^ ^15^, ^16^, ^17^, ^18^ ^]^ However, the pyrolysis process completely destroys the intriguing structural and compositional properties of the precursors, and results in limited active sites and hindered reactant transport. To take full advantage of the unique properties of MOFs, it is reasonable to directly use them as (pre)electrocatalysts. However, MOF materials suffer from unsatisfactory catalytic activity, mainly due to their intrinsic poor charge/mass transportation ability.^[^ ^19^, ^20^, ^21^ ^]^

The intrinsic low electric conductivity of MOFs limits efficient charge (electron) transfer. To address this problem, direct growth and incorporation of MOF nanosheets on conducting scaffold have been exploited for electrocatalysis.^[^ ^20^ ^]^ Metal oxide and chalcogenides nanoparticles formed in MOFs via post‐synthesis process could efficiently enhance the conductivity of MOFs and electrocatalytic performance.^[^ ^22^, ^23^ ^]^ Meanwhile, MOFs with high electric conductivity are also developed by employing *π*‐conjugated aromatic ligands.^[^ ^24^, ^25^ ^]^ The mass transport limitation in MOFs originates from the incompatible pore size of MOFs for reactants diffusion. It leads to low exposure and inadequate utilization of active sites due to the large mass resistance. In this regard, MOFs in the form of 2D ultrathin nanosheets are highly desirable and have been successfully developed by ultrasonication‐assisted exfoliation, liquid–liquid interfacial growth, organic ligand confined synthesis, sublimation‐vapor phase transformation, epitaxial growth, sacrificial template growth, and exhibited impressive electrocatalytic performances.^[^ ^26^, ^27^, ^28^, ^29^, ^30^, ^31^ ^]^

Introducing heterogeneous species into the pristine MOFs is another brilliant way to boost the electrocatalytic activity of MOFs. The conductivity and electrocatalytic performance of MOFs are enhanced by post‐synthesis plasma treatment to generate tiny CuS or CoO*_x_* nanoparticles in MOFs.^[^ ^22^, ^23^ ^]^ A partial phosphorization strategy is reported to generate CoP species within Co‐based MOF for HER catalysis.^[^ ^32^ ^]^ The heterogeneous species is able to modulate MOF's nanostructure for improved surface area and accessible active sites. Most importantly, it is also anticipated to induce possible synergistic effect between different components via formation of defects, interfacial electronic coupling, and/or strain engineering,^[^ ^3^, ^33^, ^34^, ^35^, ^36^, ^37^ ^]^ offering a great chance to optimize the adsorption energy of the critical intermediates, leading to improved intrinsic catalytic activity.

Herein we report Fe/Ni‐based MOF ultrathin nanosheets with embedded metal sulfide clusters grown on nickel foam (NiFe‐MS/MOF@NF) as a highly efficient bifunctional HER/OER electrocatalyst. It is synthesized via a simple one‐step solvothermal reaction and demonstrates impressive bifunctional electrocatalytic activity (50 mA cm^−2^ at 230 mV for OER and 50 mA cm^−2^ at 156 mV for HER). Detailed investigation reveals that the presence of Fe/Ni sulfide clusters is critical to the electrocatalytic performance; they promote the formation of ultrathin nanosheets, and efficiently improve the conductivity of the nanosheets and most importantly, they modulate the electronic structure and surface properties of the electrocatalyst, together to boost the electrocatalytic activity.

## 2 Results and Discussion

**Scheme 1:**
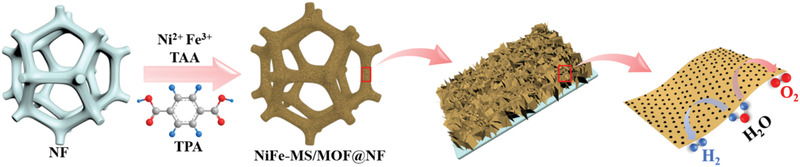
Scheme showing of the synthesis of NiFe‐MS/MOF@NF.

**Figure 1:**
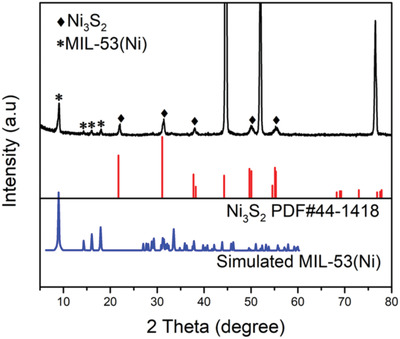
XRD pattern of NiFe‐MS/MOF@NF.

The NiFe‐MS/MOF@NF electrode was synthesized using one‐step solvothermal reaction in the presence of terephthalic acid (TPA, MOF ligand) and thioacetamide (TAA, S source) with an Fe^3+^/Ni^2+^ molar ratio of 1:4, as illustrated in **Scheme** **1**. The X‐ray powder diffraction (XRD) pattern of as‐prepared NiFe‐MS/MOF@NF (**Figure** **1**) exhibits several characteristic diffraction peaks from heazlewoodite (Ni_3_S_2_, JCPDF no. 44‐1418), a highly conductive metal sulfide with continuous network of Ni—Ni bonds.^[^ ^38^, ^39^, ^40^, ^41^ ^]^ Other diffraction peaks at 8.9°, 14.2°, 16°, and 18° could be assigned to MIL‐53 type MOF structure, respectively (no. 985 792, space group of C2/m, Cambridge Crystallographic Data Centre).^[^ ^42^, ^43^ ^]^ This XRD pattern suggests the coexistence of metal sulfide and MIL‐53 MOF in as‐prepared NiFe‐MS/MOF@NF.

**Figure 2:**
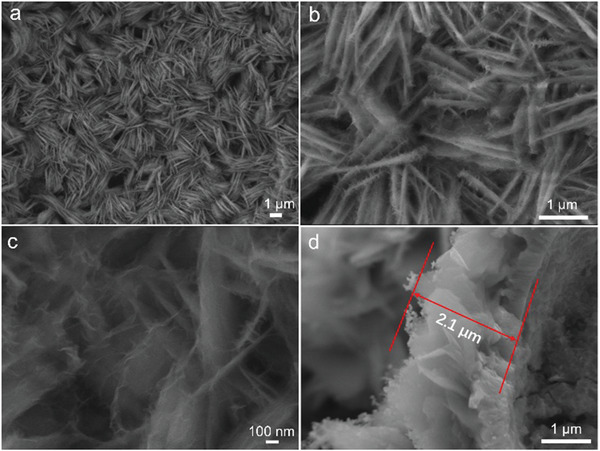
SEM images of NiFe‐MS/MOF@NF.

After solvothermal reaction, the surface of NiFe‐MS/MOF@NF is completely brownish black in color without any patches (Figure S1, Supporting Information). As shown as the scanning electron microscopy (SEM) image in **Figure** **2**a, the surface of NF is covered with dense and uniform nanosheets, which are all vertically oriented on the NF skeleton. The high‐resolution SEM images further unveil the hierarchical structure of the nanosheet array (Figure 2b,c). By interconnecting and intersecting with each other, the nanosheets (≈20–30 nm in thickness) form 3D honeycomb framework with plenty of void spaces, where there are many ultrathin nanosheets (thickness less than 10 nm) to connect these thick nanosheets. The height of the nanosheet array is ≈2.1 µm (Figure 2d). This unique open and hierarchical porous structure is beneficial to the mass transport and offers large specific surface area for expediting the electrochemical reactions.^[^ ^33^, ^44^, ^45^, ^46^ ^]^

**Figure 3:**
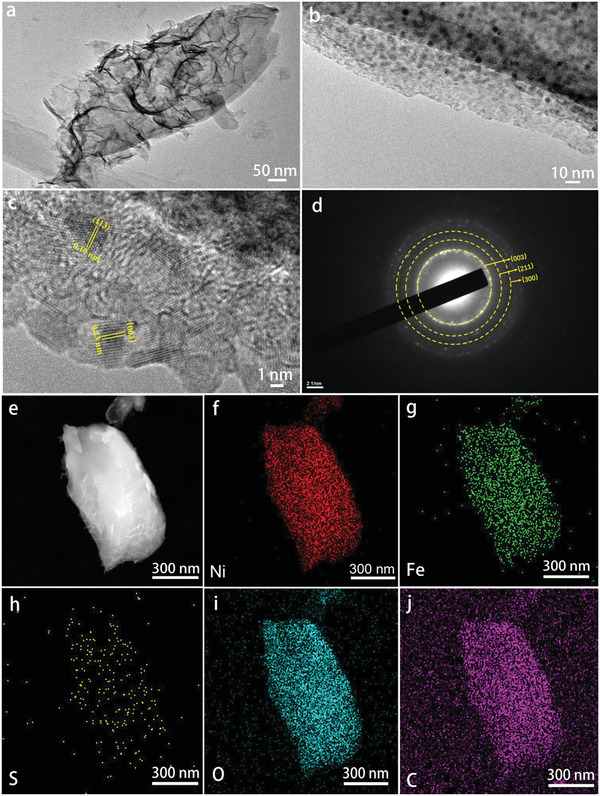
a–c) TEM images, d) SAED pattern, e) SEM image, and f) corresponding elemental Ni, g) Fe, h) S, i) O, and j) C mapping images of NiFe‐MS/MOF nanosheet.

On transmission electron microscopy (TEM) image in **Figure** **3**a, an individual NiFe‐MS/MOF nanosheet shows a lateral size of 1–2 µm, assembled by several ultrathin nanosheets. The ultrathin nanosheets show wrinkled and crumpled structure and form a porous assembly. In the ultrathin nanosheets there are dense 3–5 nm nanoclusters embedded (Figure 3b) and there are no obvious cracks on the nanosheet, implying good mechanical and electrical contacts between different components. Two lattice fringes with the d‐spacing of 0.23 and 0.19 nm are observed on the clusters, corresponding to the (003) and (113) lattice of Ni_3_S_2_ (Figure 3c), in accordance with the XRD pattern in Figure 1. The corresponding selected‐area electron diffraction (SAED) pattern (Figure 3d) displays several bright rings, which can be indexed to the (003), (211), and (300) planes of Ni_3_S_2_ crystals. No lattice fringe or diffraction pattern from MIL‐53 is observed, possibly because the MOF crystalline structure is destroyed under the high‐energy electron flow.^[^ ^27^, ^43^ ^]^

The SEM image (Figure 3e) and corresponding EDX mapping images (Figure 3f–j) of an individual NiFe‐MS/MOF nanosheet show that Ni, Fe, and C are uniformly distributed throughout the whole nanosheet, while the S element shows discrete distribution, suggesting the presence of metal sulfide clusters in the nanosheet. The EDX spectrum (Figure S2, Supporting Information) reveals an Ni/Fe atomic ratio of close to 7.5:1 in the nanosheet, which slightly differs with the Ni/Fe ratio used for solvothermal reaction and could be attributed to the different coordination ability of Ni^2+^ and Fe^3+^ in the MOF growth.^[^ ^47^ ^]^

The formation mechanism of such metal sulfide cluster embedded MOF ultrathin nanosheets is investigated. Control experiments reveal that in the absence of TAA (S source), the product (denoted as NiFe‐MOF@NF) exhibits the characteristic peaks from MIL‐53 MOF without any peaks from metal sulfide (Figure S3, Supporting Information). Pure metal sulfide (denoted as (Fe)‐Ni_3_S_2_@NF), however is obtained in the presence of TAA but absence of TPA (organic ligand, Figure S3, Supporting Information). In this case the TAA as a vulcanization reagent releases S^2−^ ions and forms metal sulfide under solvothermal condition.^[^ ^38^ ^]^ Moreover, the diffraction peak intensity from metal sulfide shows a positive correlation with the TAA dosage at a constant TPA dosage (Figures S3,S4, Supporting Information), implying that the sulfide content in the nanosheets could be well adjusted by TAA dosage. It is also noted that the NiFe‐MOF@NF shows considerably thicker nanosheet while the (Fe)‐Ni_3_S_2_@NF nanosheets are ultrathin (Figure S5, Supporting Information). Recent work demonstrates successful synthesis of ultrathin MOF nanosheets by using ultrathin LDH nanosheets as metal source as well as template under solvothermal condition.^[^ ^26^ ^]^ In present case, the diffraction peaks from MOF are considerably weak while these from sulfide are strong if a short growth time is applied (Figure S6a, Supporting Information). Therefore, it is hypothesized that the S^2−^ ions released by TAA react with metal (Ni, Fe) ions to form ultrathin sulfide nanosheets, which in turn are transformed in the presence of TPA ligand to metal sulfide cluster embedded MOF ultrathin nanosheets.^[^ ^41^ ^]^

**Figure 4:**
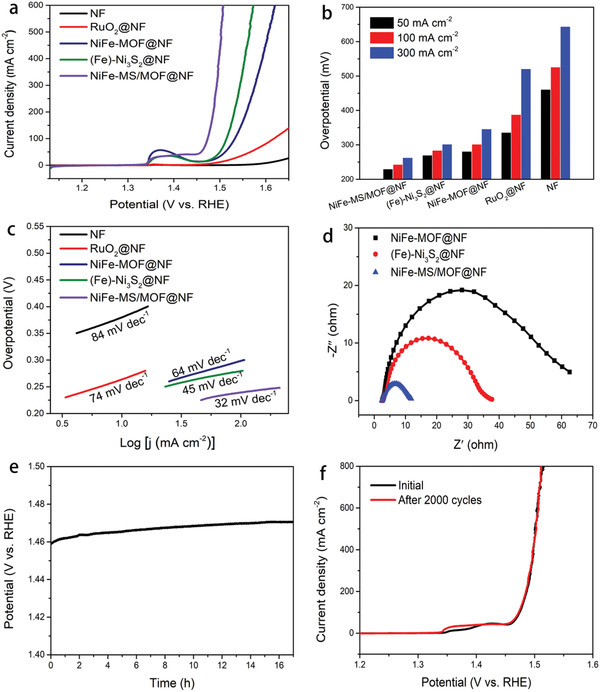
OER performance of NiFe‐MS/MOF@NF and other electrocatalysts in 1 m KOH. a) *iR*‐corrected polarization curves (scanning rate: 2 mV s^−1^), b) overpotentials required at given current density, c) Tafel plots, and d) impedance spectra; e) chronopotentiometric curve at 50 mA cm^−2^ and f) LSV curves (scanning rate: 2 mV s^−1^) before and after 2000 CV of NiFe‐MS/MOF@NF.

The NiFe‐MS/MOF@NF catalyst synthesized with 1:4 of Fe/Ni ratio for 5 h reaction shows the best OER activity among a serial of bimetal and single‐metal catalysts (Figures S6b,S7–S9, Supporting Information). The OER performance of this optimal NiFe‐MS/MOF@NF is further evaluated. According to the linear sweep voltammetry (LSV) curves in **Figure** **4**a, NiFe‐MS/MOF@NF shows impressive OER activity in alkaline media and significantly outperforms NiFe‐MOF@NF, (Fe)‐Ni_3_S_2_@NF, RuO_2_ supported on NF and bare NF, as highlighted by its lower onset potential and much higher current density at a given OER potential. NiFe‐MS/MOF@NF is able to generate a current density of 50 mA cm^−2^ at 1.46 V versus reversible hydrogen electrode (RHE), corresponding to a low overpotential of only 230 mV, as shown in Figure 4b. To achieve the same current density, the overpotentials required for (Fe)‐Ni_3_S_2_@NF, NiFe‐MOF@NF, RuO_2_@NF, and NF are 270, 281, 336, and 461 mV, respectively. The current density on NiFe‐MS/MOF@NF increases to 100 and 300 mA cm^−2^ at the overpotential of 243 and 263 mV, both far lower than those on (Fe)‐Ni_3_S_2_@NF, NiFe‐MOF@NF, RuO_2_@NF, and NF, respectively (Figure 4b). NiFe‐MS/MOF@NF electrode shows the lower Tafel slope (32 mV dec^−1^) than (Fe)‐Ni_3_S_2_@NF, NiFe‐MOF@NF, RuO_2_@NF, and NF, indicating more favorable OER kinetics, as in Figure 4c.^[^ ^48^ ^]^ As shown as the electrochemical impedance spectra (EIS) in Figure 4d, at the potential of 1.49 V, the charge‐transfer resistance (*R*_ct_) values of NiFe‐MS/MOF@NF is remarkably smaller than that of (Fe)‐Ni_3_S_2_@NF and NiFe‐MOF@NF, which agrees well with the LSV curves, indicating fast OER kinetics of NiFe‐MS/MOF@NF compared to other two electrocatalysts. These comparisons unambiguously reveal that the incorporation of sulfide cluster into the MOF nanosheet is able to essentially boost the OER activity of the resultant NiFe‐MS/MOF@NF electrocatalyst.

NiFe‐MS/MOF@NF shows excellent OER durability as well. As shown in Figure 4e, at a constant OER current density of 50 mA cm^−2^, the potential on NiFe‐MS/MOF@NF exhibits negligible increase (≈11 mV) over 17 h continuous electrolysis. Meanwhile, its polarization curve almost keeps unchanged even after accelerated durability test (ADT) of 2000 potential cycling between 1.05 and 2.05 V versus RHE (Figure 4f), showing excellent durability.

**Figure 5:**
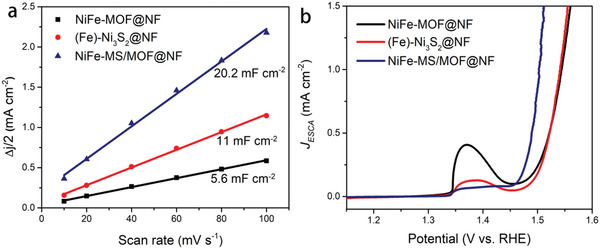
a) Electrochemical double‐layer capacitance and b) ECSA‐normalized LSV curves of NiFe‐MS/MOF@NF, (Fe)‐Ni_3_S_2_@NF, and NiFe‐MOF@NF.

In order to investigate the intrinsic OER activity of NiFe‐MS/MOF@NF, the electrochemical surface areas (ECSAs) of NiFe‐MS/MOF@NF, (Fe)‐Ni_3_S_2_@NF, and NiFe‐MOF@NF are compared by measuring their double‐layer capacitance (*C*_dl_) with CV curves at different scan rates (Figure S10, Supporting Information, calculation details provided in the caption).^[^ ^45^, ^49^ ^]^ As shown in **Figure** **5**a, the *C*_dl_ value of NiFe‐MS/MOF@NF is 1.84‐ and 3.6‐fold as that of (Fe)‐Ni_3_S_2_@NF and NiFe‐MOF@NF, respectively, suggesting higher ECSA of NiFe‐MS/MOF@NF, which agrees well with the SEM and TEM observation.

According to the ECSA‐normalized LSV curves (Figure 5b), the NiFe‐MS/MOF@NF shows lowest onset potential and highest ECSA‐normalized current density at a given potential among three electrocatalysts, indicating its higher intrinsic OER activity compared to (Fe)‐Ni_3_S_2_@NF and NiFe‐MOF@NF. Therefore the excellent OER activity of NiFe‐MS/MOF@NF is not only attributed to its large ECSA, but also is tightly associated with its high intrinsic OER activity, which is believed to originate from the synergistic effect between the MOF nanosheets and embedded sulfide clusters.

To further elucidate the possible mechanism behind this synergistic effect, the structural and compositional evolution of NiFe‐MS/MOF@NF after OER is examined. After OER test (typically 20 cycles between 1.1 and 2.1 V vs RHE at 50 mV s^−1^), the diffraction peaks from MOF completely disappear and these of metal sulfide have no obvious change, as shown in XRD pattern in Figure S11a, Supporting Information, suggesting that the crystalline structure of MOF sheets have transformed during the OER while the sulfide clusters survive, which is confirmed by the Raman spectra (Figure S11b, Supporting Information).^[^ ^27^ ^]^ The sulfide clusters survived possibly because they were protected by the surrounding oxyhydroxide matrix from phase change during OER process. The nanosheet morphology of the electrocatalyst keeps unchanged but the thickness considerably increases (Figure S12, Supporting Information), suggesting the formation of disordered oxyhydroxides upon OER testing, which serves as real active sites for water oxidation.^[^ ^13^, ^20^, ^50^ ^]^

**Figure 6:**
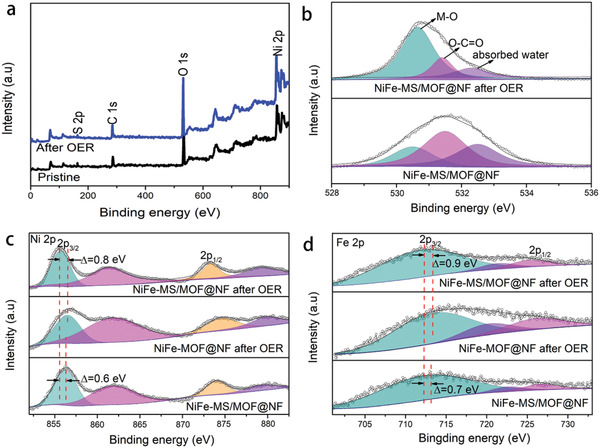
a) XPS survey spectra and b) O 1s spectra of NiFe‐MS/MOF@NF before and after OER test. Inset in (a) shows the corresponding elemental percentages; c) Ni 2p and d) Fe 2p spectra of NiFe‐MS/MOF@NF before and after OER test and of NiFe‐MOF@NF after OER test.

The electronic interaction in NiFe‐MS/MOF@NF is probed by X‐ray photoelectron spectroscopy (XPS) measurement. The survey spectrum in **Figure** **6**a confirms the presence of Ni, Fe, S, O, and C elements in the pristine nanosheets (element content is listed in Table S1, Supporting Information), and the atomic ratio of Fe/Ni is found to be 1:8, which agrees well with EDX results. After OER test, the atomic percentages of Ni, Fe, and S do not change significantly but that of C considerably decreases, possible due to the leakage of organic ligand. In O 1s spectrum (Figure 6b), the NiFe‐MS/MOF@NF shows three subpeaks corresponding to the O binding to metal (M—O), O in carboxyl group (O=C—O—), and O from absorbed water. After OER test, the percentage of carboxyl O evidently decreases while the M—O increases, again suggesting the conversion of MOF to oxyhydroxides during OER. Notably, the binding energy of Ni 2p_3/2_ in NiFe‐MS/MOF@NF decreases after OER test (ΔBE = 0.6 eV, Figure 6c). Meanwhile, its binding energy is also evidently lower than that of NiFe‐MOF@NF after OER test (ΔBE = 0.8 eV). We note that all the samples were scanned to resting potential before the XPS measurement. Similar binding energy shift is also observed in Fe 2p spectrum (Figure 6d). It implies that after OER, the metal sites in NiFe‐MS/MOF@NF significantly change their electronic structure to electron‐rich state. This is because the metal sulfide clusters endow electrons to the Ni and Fe centers on the surface from their electron rich metal atoms (as indicated by the XPS spectra of pristine Fe‐(Ni_3_S_2_)@NF shown in Figure S13, Supporting Information).^[^ ^51^ ^]^ Possible strain effect may also contribute to the charge transfer process.^[^ ^33^ ^]^

Ni/Fe‐based catalysts are identified as the best alkaline OER electrocatalysts to date.^[^ ^52^, ^53^ ^]^ The active site is proposed to be the di‐µ‐oxo bridged Ni—Fe atoms in the in situ generated Ni/Fe oxyhydroxide surface layer, where Fe stabilizes the formed O radicals while Ni catalyzes the subsequent O—O coupling.^[^ ^54^, ^55^ ^]^ Recent research further suggests that the OER activity of Ni/Fe‐based catalyst could be enhanced by strengthening the adsorption of oxygenated intermediates such as *OH and *O on the surface.^[^ ^54^, ^56^, ^57^ ^]^ Fe/Ni centers at electron‐rich state are conducive to enhance the oxygenated intermediates for accelerated OER kinetics,^[^ ^58^, ^59^, ^60^ ^]^ which is confirmed by the lower Tafel slope and smaller *R*_ct_ of NiFe‐MS/MOF@NF compared to NiFe‐MOF@NF in present work as shown in Figure 4c,d. Therefore, the higher intrinsic OER activity of NiFe‐MS/MOF@NF originates from the higher electron density on its metal sites, as a consequence of incorporation of metal sulfide clusters.

**Figure 7:**
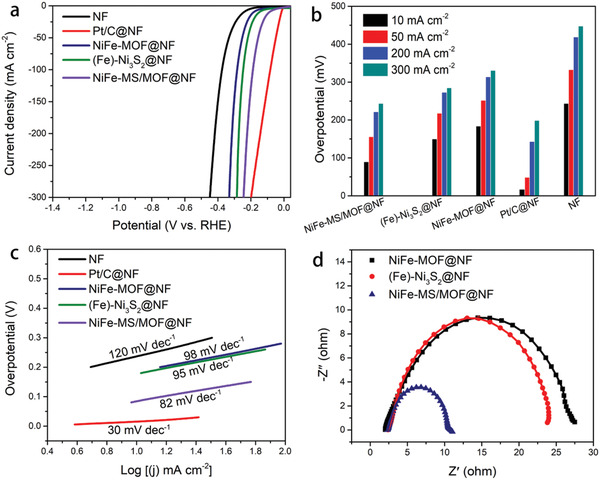
a) *iR*‐corrected HER polarization curves, b) overpotentials required at given current density, c) Tafel plots and d) impedance spectra of NiFe‐MS/MOF@NF and other electrodes in 1 m KOH. LSV scanning rate: 2 mV s^−1^.

Remarkably, this NiFe‐MS/MOF@NF electrocatalyst also demonstrates better HER activity in alkaline than its counterparts synthesized with different Fe/Ni ratios (Figure S14, Supporting Information). It also surpasses (Fe)‐Ni_3_S_2_@NF and NiFe‐MOF@NF, even though is inferior to commercial Pt/C, as shown in **Figure** **7**a. It needs an overpotential of only 90, 156, 222, and 244 mV to support HER current density of 10, 50, 200, and 300 mA cm^−2^, respectively, all of which are considerably lower than that required on NiFe‐MOF@NF, (Fe)‐Ni_3_S_2_@NF, and bare NF (Figure 7b). The Tafel slope of NiFe‐MS/MOF@NF is 82 mV dec^−1^, lower than NiFe‐MOF@NF (98 mV dec^−1^) and (Fe)‐Ni_3_S_2_@NF (95 mV dec^−1^), as shown in Figure 7c, suggesting a fast HER kinetics via Volmer–Heyrovsky route.^[^ ^61^ ^]^ On the Nyquist plots (Figure 7d), NiFe‐MS/MOF@NF shows smallest charge transfer resistance as well. The enhanced HER activity may be associated with the modulated binding strength of *OH in alkaline solution.^[^ ^62^, ^63^ ^]^ At a current density of 50 mA cm^−2^, the overpotential on NiFe‐MS/MOF@NF shows negligible increase over 28 h continuous electrolysis, as presented in Figure S15a, Supporting Information, exhibiting excellent HER stability. Notably, NiFe‐MS/MOF@NF remains the nanosheet morphology (Figure S15b, Supporting Information) but its MOF crystalline structure disappears upon HER durability test (Figure S16, Supporting Information). Previous investigation has revealed that during HER, the *M*O_6_ (*M* = Fe, Ni) units in the MOF could be partially reduced to form disordered *M*/*M*O_6_ interface and the produced OH^−^ could displace the organic ligand, resulting in the transformation from MOF to metal hydroxide.^[^ ^20^, ^50^ ^]^ Therefore, the real HER active material of NiFe‐MS/MOF@NF is not MOF itself, but in situ formed hydroxide derivatives.

**Figure 8:**
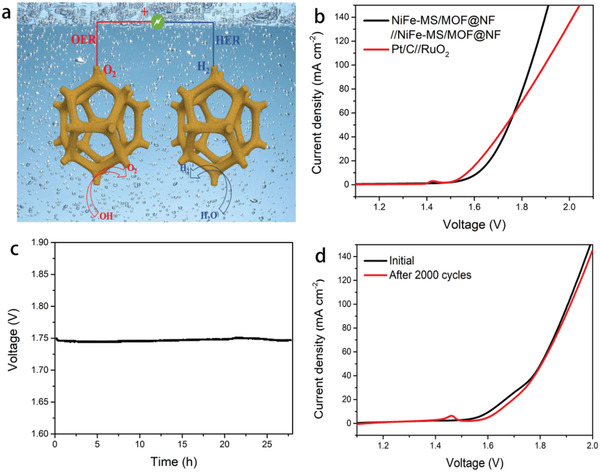
Schematic illustration of a) the electrolyzer using NiFe‐MS/MOF@NF as bifunctional electrocatalyst, b) polarization curves (without *iR* compensation) of NiFe‐MS/MOF@NF‐based and Pt/C‐RuO_2_‐based electrolyzers, scan rate 2 mV s^−1^, c) voltage–time curve at a constant current density of 50 mA cm^−2^ and d) polarization curves before and after 2000 cycles between 1–2 V at a scan rate of 50 mV s^−1^ of NiFe‐MS/MOF@NF‐based electrolyzer.

NiFe‐MS/MOF@NF is further utilized as both anode and cathode for overall water splitting in 1 m KOH (**Figure** **8**a). It needs low cell voltages of 1.61 and 1.74 V without *iR* compensation to afford an electrolytic current density of 10 and 50 mA cm^−2^, respectively (Figure 8b). Compared to the electrolyzer equipped with state‐of‐the‐art electrocatalysts, namely, Pt/C for HER and RuO_2_ for OER, this NiFe‐MS/MOF@NF‐based electrolyzer shows slightly lower current density at low cell voltage range but surpasses its counterpart at high voltage range, reflecting its excellent performance for high current density electrolysis. It also shows a superb durability as only negligible voltage augment is observed over 27 h continuous electrolysis at a constant current density of 50 mA cm^−2^ (Figure 8c). The polarization curve only shifts slightly after 2000 potential cycling between 1–2 V (Figure 8d), further highlighting the outstanding stability of NiFe‐MS/MOF@NF for both HER and OER in alkaline. The calculated Faraday efficiency (FE%) is ≈98% by measuring the generated O_2_ and H_2_ with water drainage method after 1 h electrolysis in an H‐type electrolytic cell (Figure S17, Supporting Information).^[^ ^64^ ^]^ Compared to other reported non‐precious metal based bifunctional electrocatalysts, as‐reported NiFe‐MS/MOF@NF shows very competitive electrocatalytic performance as listed in Table S2, Supporting Information.

## 3 Conclusions

In summary, we successfully synthesized NiFe‐MS/MOF@NF, made up of vertically aligned interconnected MOF ultrathin nanosheets with embedded metal sulfide clusters on NF, via a simple one‐step solvothermal reaction and demonstrated its excellent OER and HER activity in alkaline. It exhibits excellent electrocatalytic activity and durability toward both OER and HER. As a bifunctional electrocatalyst, it enables stable alkaline water electrolysis of 10 and 50 mA cm^−2^ over 27 h at a cell voltage of 1.61 and 1.74 V, respectively. The presence of metal sulfide clusters is found to be critical as they facilitate the formation of ultrathin MOF nanosheets and improve the electrical conductivity. Most importantly, the sulfide clusters are able to tune the adsorption energy of oxygen‐containing intermediates on the active metal sites in in situ formed amorphous oxyhydroxides layer, thus to significantly enhance the intrinsic electrocatalytic activity. This work offers useful insights into the rational design of MOF‐derived hybrid materials for electrocatalysis application.

## 4 Experimental Section

##### Materials and Reagents

NF (thickness: ≈1.0 mm, areal density: ≈300g m^2^) was purchased from Taiyuan Liyuan Co., Ltd. Ni(NO_3_)_2_, FeCl_3_, TPA, *N*,*N*‐dimethylformamide (DMF), TAA, and ethanol were obtained from Sinopharm Chemical Reagent Co. Ltd. (Shanghai, China) and used without further purification. KOH of semiconductor grade (99.99% trace metals basis) was purchased from Sigma‐Aldrich.

##### Synthesis of NiFe‐MS/MOF@NF

A piece of NF (4 × 2.8 cm^2^) was ultrasonically treated in acetone and 1 m HCl solution, followed by washing with distilled water. In synthesis of NiFe‐MS/MOF@NF, TPA (0.8 mmol, 133 mg), Ni(NO_3_)_2_·6H_2_O (0.768 mmol, 223.3 mg), FeCl_3_·6H_2_O (0.192 mmol, 51.9 mg), and TAA (1.33 mmol, 100 mg) were added to 20 mL of DMF and 8 mL ethanol in a beaker, followed by stirring for 20 min to obtain a homogeneous solution. The solution was transferred to a stainless steel autoclave and a piece of pretreated NF was immersed into the solution. The autoclave was sealed and maintained at 150 °C for 5 h. The resulting product was thoroughly rinsed with distilled water, and then dried at 60 °C overnight to obtain NiFe‐MS/MOF@NF. For comparison, other NiFe‐MS/MOF@NF electrocatalysts were also synthetized using different Fe/Ni ratio while maintaining a total metal amount of 0.96 mmol.

##### Synthesis of NiFe‐MOF@NF

NiFe‐MOF@NF was synthesized using the same procedure as that for NiFe‐MS/MOF@NF described above except for no TAA added in the solution.

##### Synthesis of (Fe)‐Ni_3_S_2_@NF

(Fe)‐Ni_3_S_2_@NF was synthesized using the same procedure as that for NiFe‐MS/MOF@NF described above except for no TPA added in the solution.

##### Fabrication of Pt/C@NF and RuO_2_@NF Electrodes

10 mg of Pt/C (20 wt%) or RuO_2_ powder (99.9%) was dispersed in the mixture of ethanol (985 µL) and Nafion solution (5 wt% 15 µL), respectively. After ultrasonic treatment for 30 min to obtain a homogeneous ink, 250 µL of ink was loaded on the NF (1 cm × 1 cm) and dried at 60 °C. The loading amount of Pt/C and RuO_2_ catalysts was estimated to be 2.5 mg cm^−2^.

##### Material Characterizations

SEM images were collected on a field emission SEM instrument (JSM‐7800F from JEOL). TEM was carried out on a JOEL, TEM‐2100 system after ultrasonically peeling off the materials from NF. The crystal structures were probed by XRD (Shimadzu XRD‐7000 diffractometer with Cu K*α* line). XPS measurement was performed on an ESCALAB 250Xi system from Thermo Fisher.

##### Electrochemical Measurements

Electrochemical measurements were carried out on a 760E workstation (CH Instrument, USA) in three‐electrode setup in glass‐free electrochemical cell with freshly prepared 1.0 m KOH as electrolyte solution. An Hg/HgO and a graphite rod were used as the reference electrode and counter electrode, respectively. All potentials reported in this work were converted to RHE after careful calibration of the used Hg/HgO according to the well‐established method.^[^ ^65^ ^]^ The catalytic activity was tested via LSV at a scan rate of 2 mV s^−1^. Before LSV test, 20 potential cycling between 1 and 1.8 V at 50 mV s^−1^ were carried out to fully activate the electrode. All polarization curves (except for these tested in electrolyzer) were subjected *iR* correction with respect to the solution resistance according to *E* = *E*_RHE_ − *iR*, where *E* is the *iR* corrected potential, *E*_RHE_ is the measured potential with respect to RHE, *i* is the measured current, and *R* is the solution resistance. The *R* value was obtained from EIS, which was carried out in the frequency range from 0.01 Hz to 1000 kHz with 5 mV amplitude. The long‐term durability measurements were carried out by using the chronopotentiometric test and ADT.^[^ ^66^ ^]^

## Conflict of Interest

The authors declare no conflict of interest.

## Supporting information

Supporting Information
