# Supplementary material for: Strong Electronic Interaction Enhanced Electrocatalysis of Metal Sulfide Clusters Embedded Metal–Organic Framework Ultrathin Nanosheets toward Highly Efficient Overall Water Splitting
Source: Adv Sci (Weinh). 2020 Sep 21;7(20):2001965. doi: 10.1002/advs.202001965 (PMC7578852; doi:10.1002/advs.202001965)
Supplement: Supplementary file 1 — Supporting Information [file ADVS-7-2001965-s001.pdf]

© 2020 Wiley-VCH GmbH

ADVANCED SCIENCE
Open Access

Supporting Information

for *Adv. Sci.,* DOI: 10.1002/advs.202001965

Strong Electronic Interaction Enhanced Electrocatalysis of Metal Sulfide Clusters Embedded Metal-Organic Framework Ultrathin Nanosheets toward Highly Efficient Overall Water Splitting

Ming Zhao, Wei Li, Junying Li, Weihua Hu, and Chang Ming Li**

© 2020 Wiley-VCH GmbH

Supporting Information

Strong Electronic Interaction Enhanced Electrocatalysis of Metal Sulfide Clusters Embedded Metal-Organic Framework Ultrathin Nanosheets toward Highly Efficient Overall Water Splitting

Ming Zhao, Wei Li, Junying Li, Weihua Hu, and Chang Ming Li**

Dr. M. Zhao, W. Li, J. Y. Li, Prof. Dr. W. H. Hu, Prof. Dr. C. M. Li
Key Laboratory of Luminescence Analysis and Molecular Sensing (Southwest University), Ministry of Education; Institute for Clean Energy and Advanced Materials, School of Materials & Energy, Southwest University; Chongqing Key Laboratory for Advanced Materials and Technologies of Clean Energies, Chongqing 400715, China

Prof. Dr. C. M. Li
Institute of Materials Science & Devices, School of Materials Science and Engineering, Suzhou University of Science and Technology, Suzhou 215009, China; Institute of Advanced Cross-field Science, College of Life Science, Qingdao University, Qingdao 200671, China.

* Corresponding author. E-mail: whhu@swu.edu.cn (W. H. Hu); ecmli@swu.edu.cn (C. M. Li).

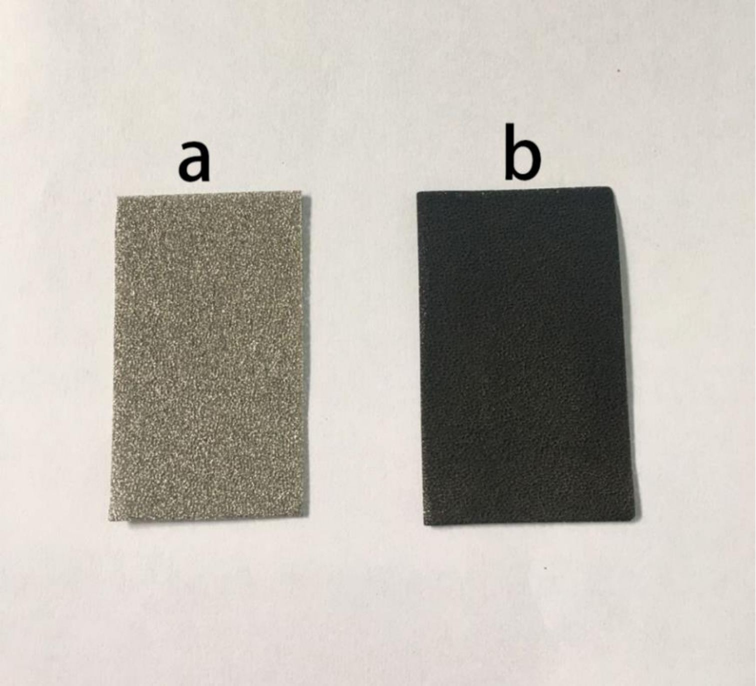

Figure S1. Photographs of NF (a) and NiFe-MS/MOF@NF (b).

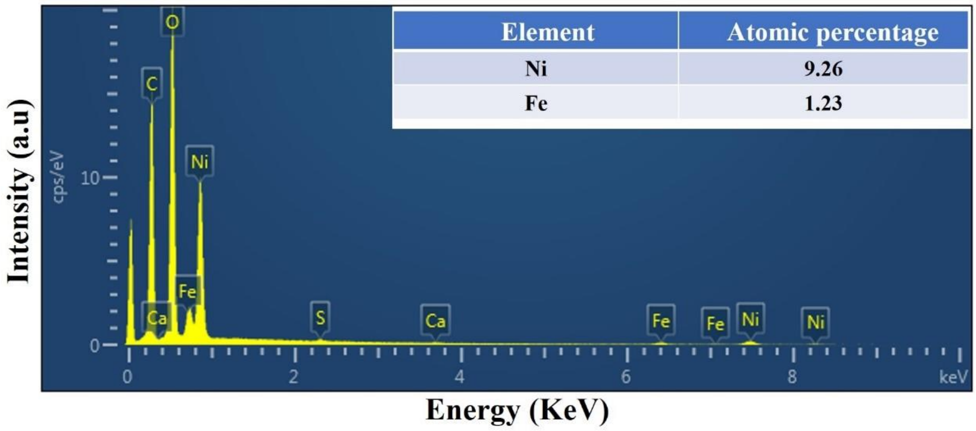

Figure S2. EDX spectrum and atomic percentages of NiFe-MS/MOF@NF nanosheets.

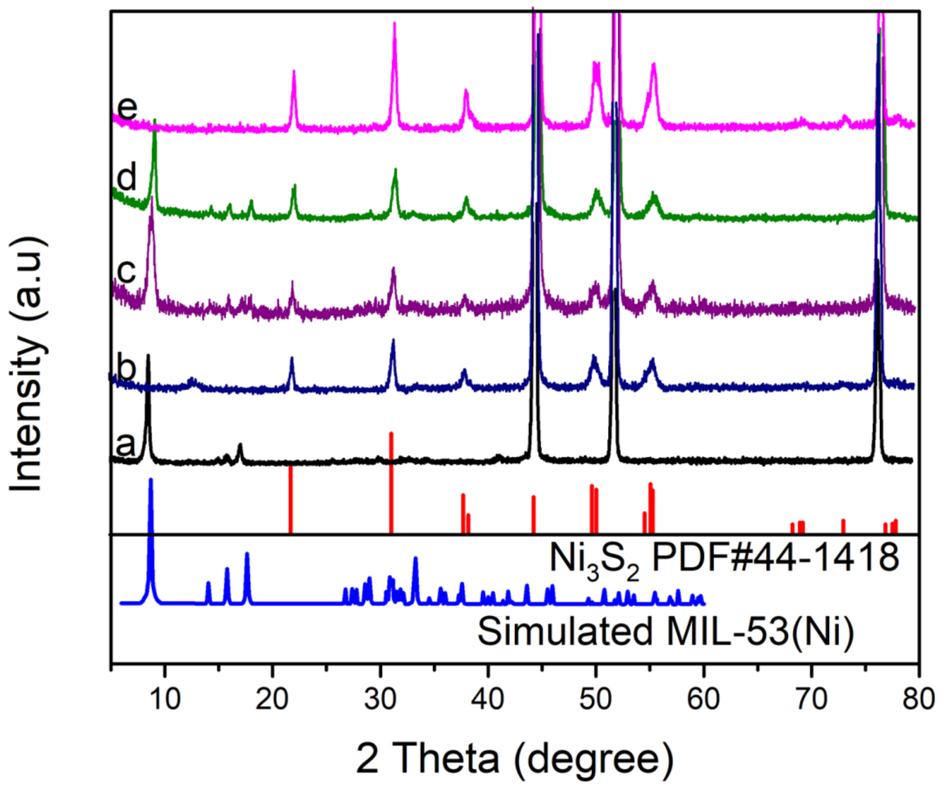

Figure S3. XRD patterns of (a) NiFe-MS/MOF@NF, (b) (Fe)-Ni_3S_2@NF and different NiFe-MS/MOF@NF samples synthesized with different mount of TAA (c) 0.05 g, (d) 0.1 g and (e) 0.15 g.

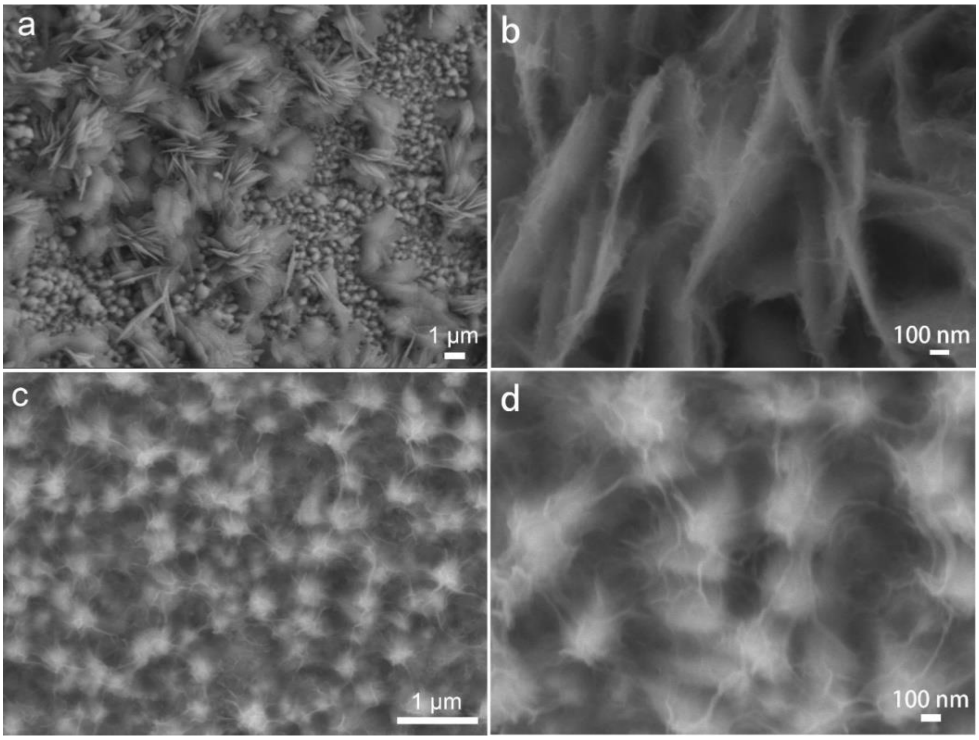

Figure S4. SEM images of different NiFe-MS/MOF@NF samples synthesized with different mount of TAA, 0.05g (a, b) and 0.15g (c, d).

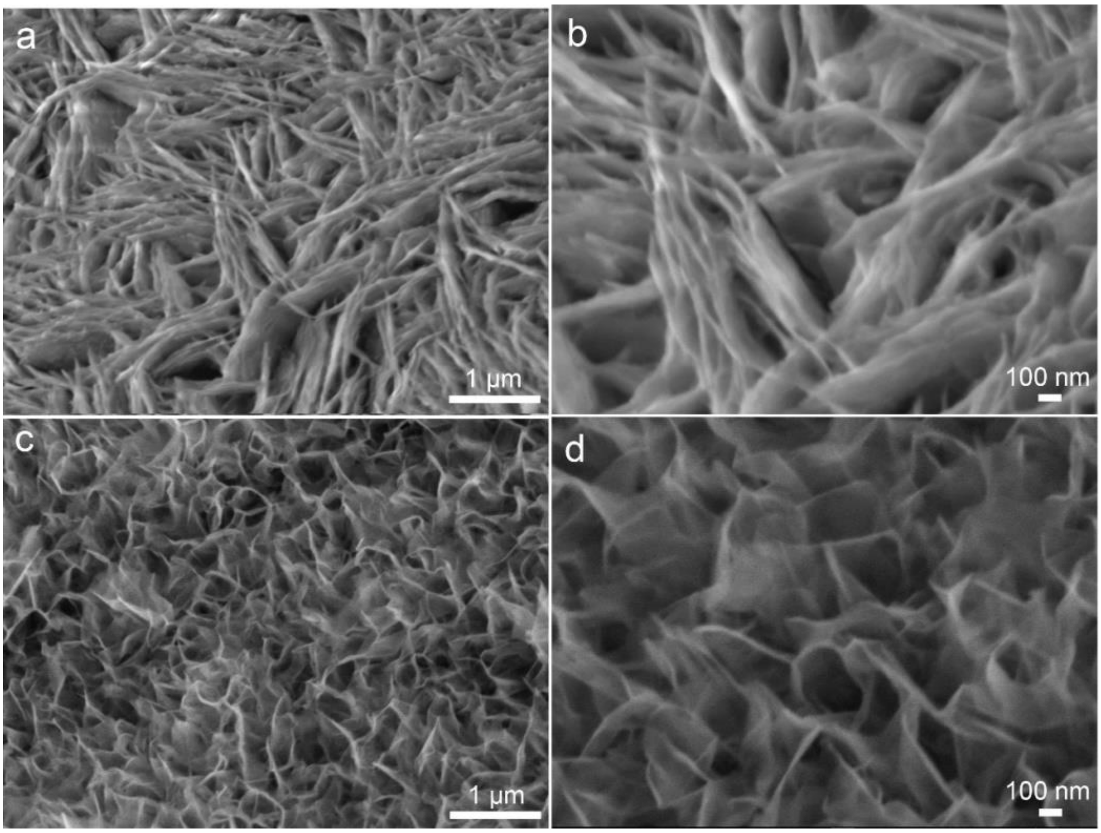

Figure S5. SEM images of NiFe-MOF@NF (a, b) and (Fe)-Ni_3S_2@NF (c, d).

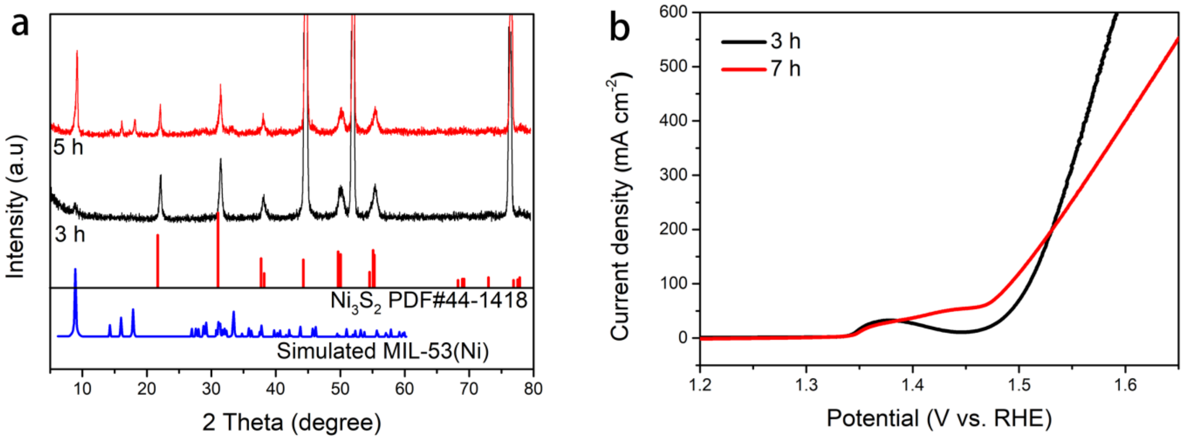

Figure S6. (a)XRD patterns of NiFe-MS/MOF@NF synthesized with 3 and 5 h reaction; (b) iR-corrected OER polarization curves of NiFe-MS/MOF@NF electrodes synthesized with 3 h and 7 h reaction time.

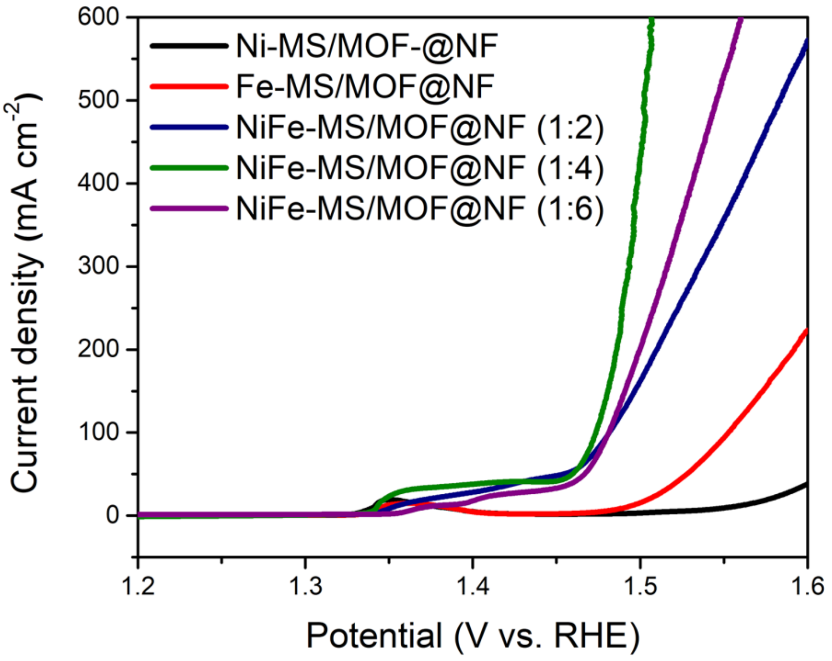

Figure S7. iR-corrected OER polarization curves of a batch of NiFe-MS/MOF@NF electrodes synthesized with different Fe/Ni dosage ratios.

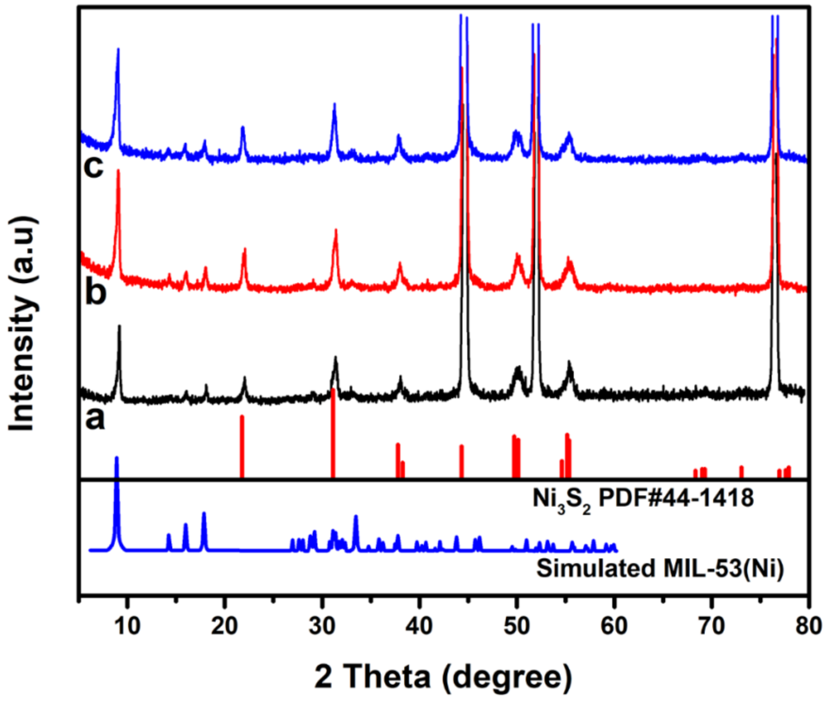

Figure S8. XRD patterns of NiFe-MS/MOF@NF samples synthesized with different Fe/Ni dosage ratio of (a) 1:2, (b)1:4, (c) 1:6.

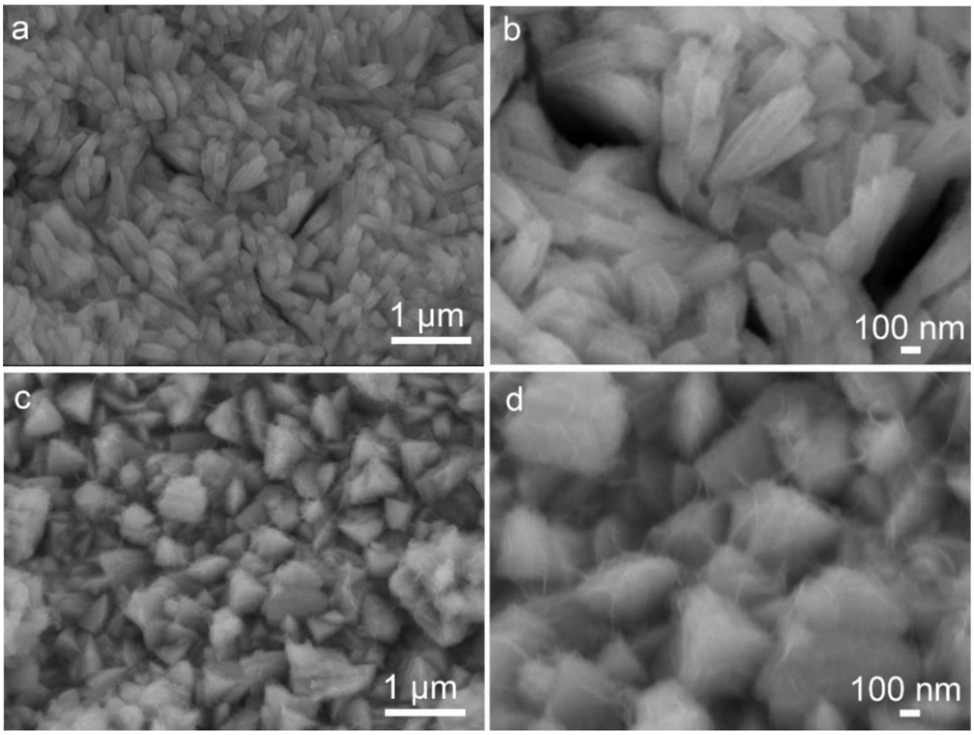

Figure S9. SEM images of NiFe-MS/MOF@NF synthesized with Fe/Ni dosage ratio of 1:2 (a,b) and 1:6 (c,d).

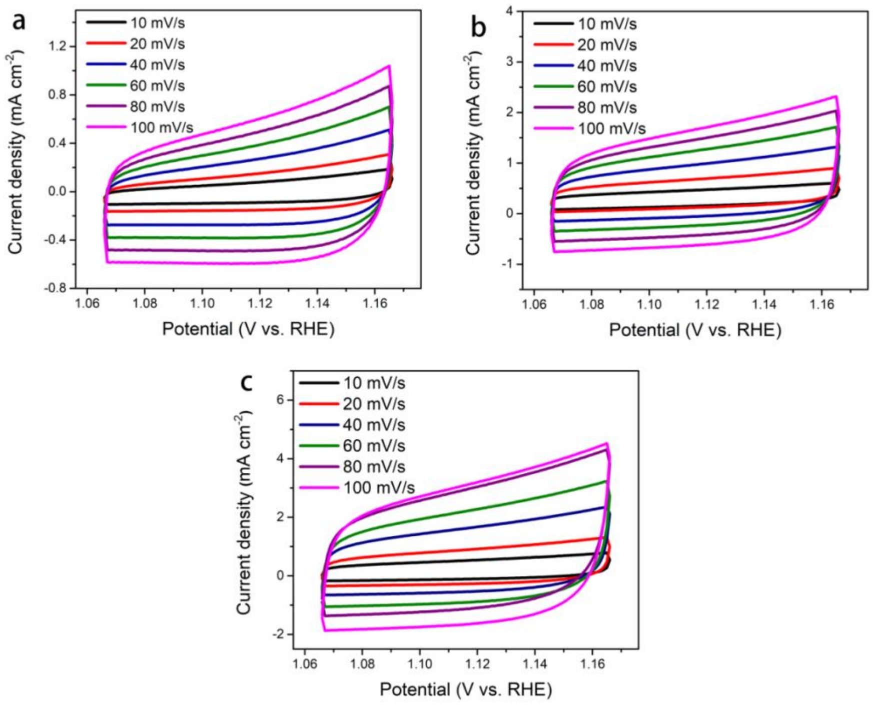

Figure S10. CV curves of NiFe-MOF@NF (a), (Fe)-Ni_3S_2@NF (b) and NiFe-MS/MOF@NF (c) with scan rates ranging from 10 to 100 mV s^{-1}. By plotting the capacitive currents (ΔJ, $(J_a-J_c)/2$) against the scanning rate and following with a linear fit, the C_{dl} was estimated as the slope. The C_{dl} can be further converted into ECSA using the specific capacitance value for a flat surface of 0.04 mF cm^{-2} according to ECSA=$\frac{C_{dl}}{0.04\ mF\ cm^{-2}}$.

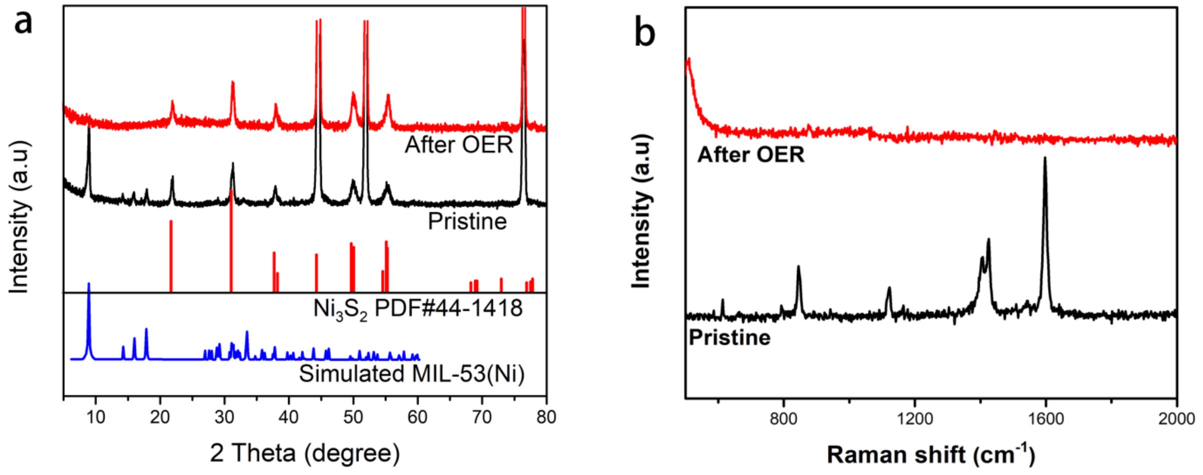

Figure S11. XRD patterns (a) and Raman spectra (b) of NiFe-MS/MOF@NF before and after OER test.

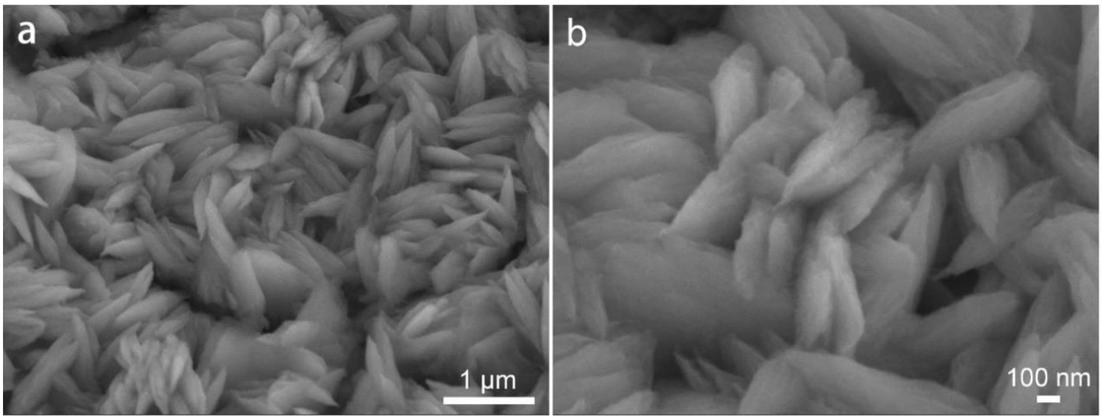

Figure S12. SEM images of NiFe-MS/MOF@NF after OER test.

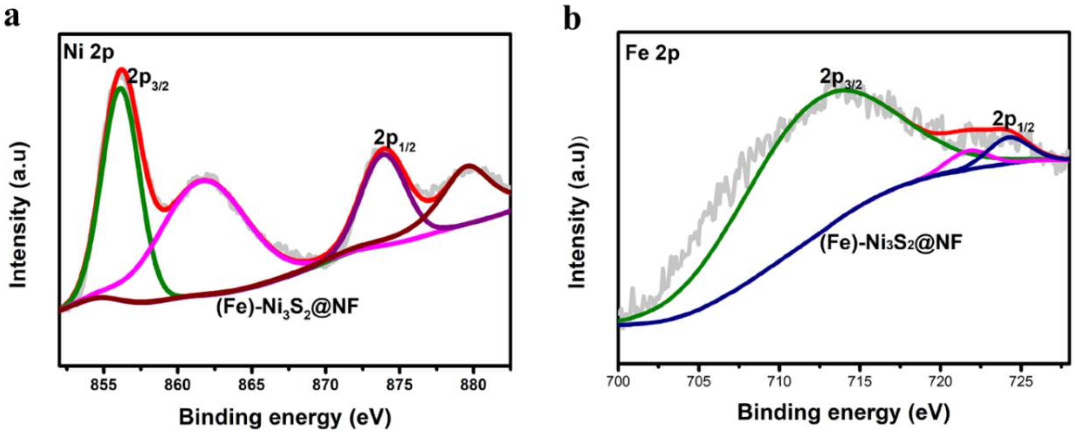

Figure S13. Ni 2p and Fe 2p XPS spectra of (Fe)-Ni_3S_2@NF.

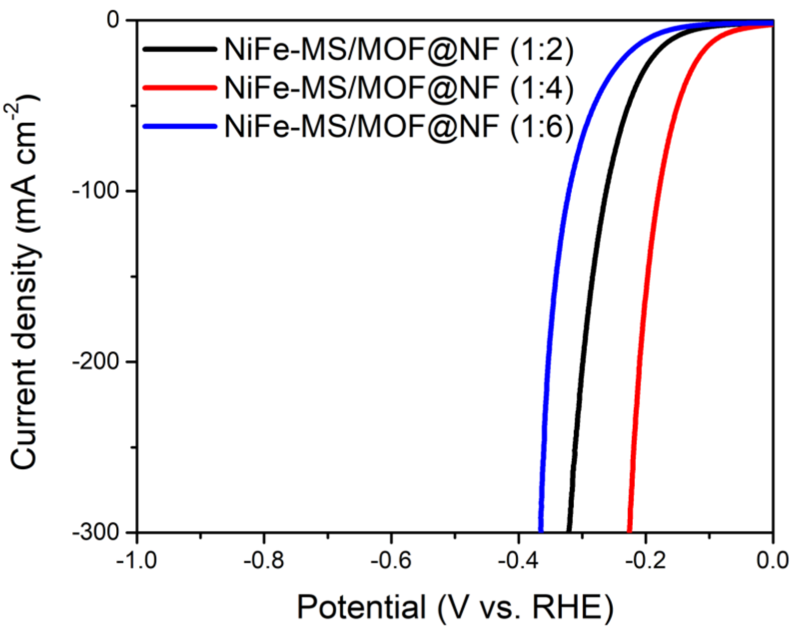

Figure S14. iR-corrected HER polarization curves of a batch of NiFe-MS/MOF@NF electrodes synthesized with different Fe/Ni dosage ratios.

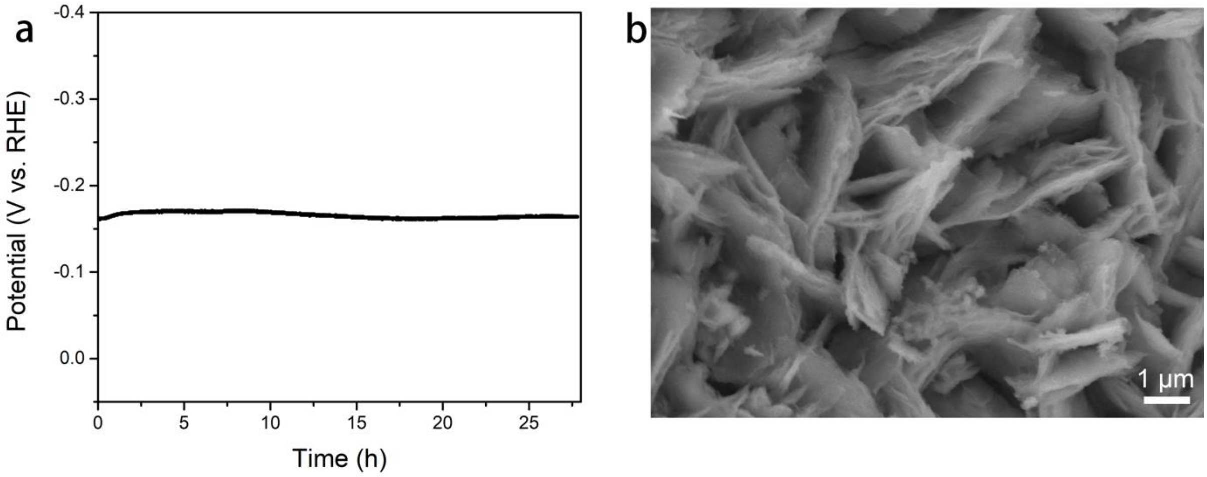

Figure S15. (a) Chronopotentiometric curve of NiFe-MS/MOF@NF at a constant HER current density of 50 mA cm^{-2}; (b) SEM image of NiFe-MS/MOF@NF after durability test.

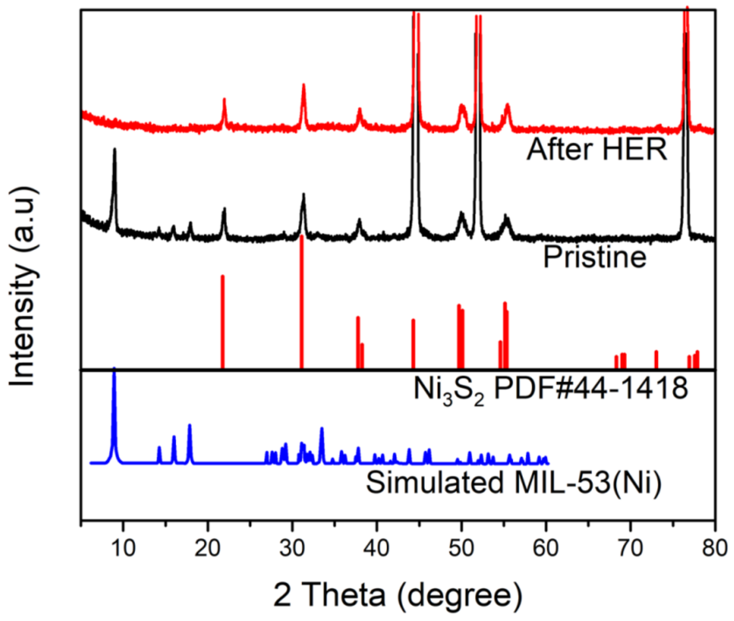

Figure S16. XRD pattern of NiFe-MS/MOF@NF after HER durability test.

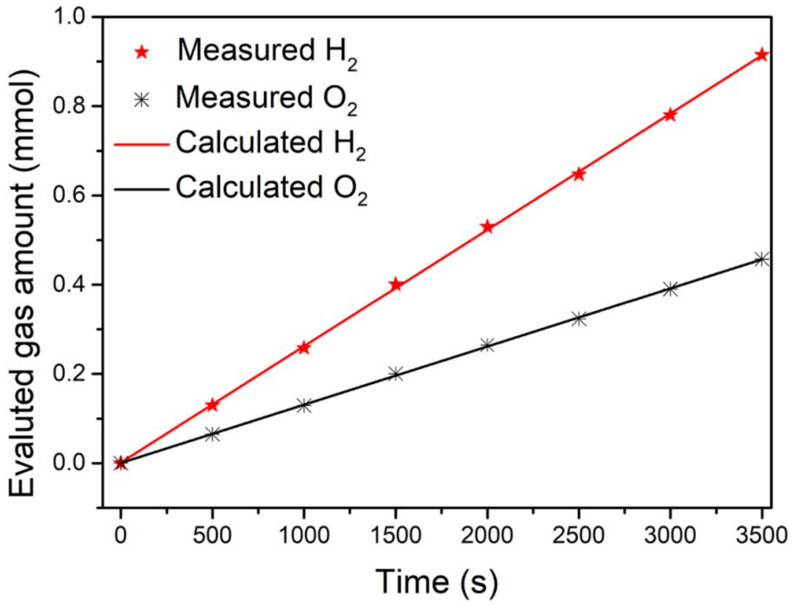

Figure S17. The volume of gas theoretically calculated and experimentally measured versus time on NiFe-MS/MOF@NF-based electrolytic cell. The Faraday efficiency (FE%) was calculated by the equation: FE= $znF/It\times100\%$, where z is the electron transfer number (z=2 for per mole H_2, z=4 for per mole O_2), n is the amount (moles) of the generated gas in the experiment, F is the Faraday constant (96485.3 C mol^{-1}), I is the applied constant current (A), and t is the electrolysis time (s). The amount of H_2/O_2 generated in the H-type electrolytic cell was collected by the water drainage method.

Table S1. Element content of NiFe-MS/MOF@NF before and after OER test, measured by XPS.

Element	at%	
	**before OER**	**after OER**
**Ni**	17.55	19.80
**Fe**	2.19	2.35
**S**	4.36	2.63
**O**	42.20	56.20
**C**	33.70	19.02

Table S2. Comparison of the electrocatalytic activity of the recently reported non-noble metal based bifunctional catalysts.

Electrode	η_{HER} (mV@mA cm^{-2})	η_{OER} (mV@mA cm^{-2})	Cell voltage (V@mA cm^{-2})	References
**NiFe-MS/MOF@NF**	156 @50	230 @50	1.74 @50	This work
**NiFeZn-MOF**	180@10	350@50	1.52@10	[1]
**MNF-MOFs/NF**	79@10	235@50	1.495@10	[2]
**NiFe/NiCo$_2$O$_4$/NF**	105@10	270@60	1.67@10	[3]
**NFN-MOF/NF**	87@10	240@10	1.56@10	[4]
**(Ni,Co)Se$_2$-GA/NF**	128@10	320@10	1.60@10	[5]
**NiFe LDH@NiCoP/NF**	120@10	220@10	1.57@10	[6]
**Fe-Ni$_3$S$_2$/NF**	47@10	214@10	1.54@10	[7]
**Ni$_3$FeN/r-GO-NF**	94@10	270@10	1.60@10	[8]
**Ni-Co-P HNBs on NF**	107@10	270@10	1.62@10	[9]
**FeMnP/GNF**	84@10	280@10	1.55@10	[10]
**Ni-Co-P HNBs on NF**	107@10	270@10	1.62@10	[9]
**NiCo$_2$S$_4$NW/NF**	210@10	260@10	1.63@10	[11]
**S-NiFe2O4/NF**	138@10	267@10	1.65@10	[12]
**MoS$_2$-Ni$_3$S$_2$ HNRs/NF**	98@10	249@10	1.50@10	[13]
**Ni/Ni$_8$P$_3$-NF**	130@10	270@30	1.61@10	[14]

[1] X. Wei, N. Li, N. Liu, *Electrochim. Acta.* **2019**, 318, 957.

[2] D. S. Raja, H. Lin, S. Lu, *Nano Energy.* **2019**, 57, 1.

[3] X. Zhang, L. Yu, Y. Gui, W. Hu, *Applied Surface Science* **2016**, 367, 259.

[4] D. S. Raja, X. Chuah, S. Lu, *Adv. Energy. Mater.* **2018**, 8, 1801065.

[5] M. C.-A. Junqiao Zhuo, Hanfeng Liang, Leith Samad, Qi Ding, Yongping Fu, Meixian Li, and Song Jin, *Acs Catalysis.* **2015**, 5, 6355.

[6] H. Zhang, X. Li, A. Hahnel, V. Naumann, C. Lin, S. Azimi, S. L. Schweizer, A. W. Maijenburg, R. B. Wehrspohn, *Adv. Funct. Mater.* **2018**, 28, 1706847.

[7] G. Zhang, Y. Feng, W. Lu, D. He, C. Wang, Y. Li, X. Wang, F. Cao, *ACS Catalysis.* **2018**, 8, 5431.

[8] Y. Gu, S. Chen, J. Ren, Y. A. Jia, C. Chen, S. Komarneni, D. Yang, X. Yao, *ACS Nano.* **2018**, 12, 245.

[9] E. Hu, Y. Feng, J. Nai, D. Zhao, Y. Hu, X. W. Lou, *Energy Environ. Sci.* **2018**, 11, 872.

[10] Z. Zhao, D. E. Schipper, A. P. Leitner, H. Thirumalai, J.-H. Chen, L. Xie, F. Qin, M. K. Alam, L. C. Grabow, S. Chen, D. Wang, Z. Ren, Z. Wang, K. H. Whitmire, J. Bao, *Nano Energy.* **2017**, 39, 444.

[11] S. Li, Y. Wang, S. Peng, L. Zhang, A. M. Al-Enizi, H. Zhang, X. Sun, G. Zheng, *Adv. Energy. Mater.* **2016**, 6, 1501661.

[12] Y. Tang, C. Yang, Y. Yang, X. Yin, W. Que, J. Zhu, *Electrochim. Acta.* **2019**, 296, 762.

[13] Y. Yang, K. Zhang, H. Lin, X. Li, H. C. Chan, L. Yang, Q. Gao, *ACS Catalysis.* **2017**, 7, 2357.

[14] G.-F. Chen, T. Y. Ma, Z.-Q. Liu, N. Li, Y.-Z. Su, K. Davey, S.-Z. Qiao, *Adv. Funct. Mater.* **2016**, 26.
